# The correlations between parental warmth and children’s approaches to learning: a moderated mediation model of self-efficacy and teacher-child closeness

**DOI:** 10.3389/fpsyg.2024.1290141

**Published:** 2024-03-18

**Authors:** Yongli Liu, Wei Wang, Sumei Wei, Pengcheng Wang, Kun Chen, Jing Liu, Junjun Chen

**Affiliations:** ^1^Department of Education Policy and Leadership, The Education University of Hong Kong, Hong Kong, Hong Kong SAR, China; ^2^Department of Educational, Shanxi Datong University, Datong, China; ^3^Department of Education, Quzhou University, Quzhou, China; ^4^School of Media and Communication, Shanghai Jiao Tong University, Shanghai, China; ^5^School of Journalism, Shanxi Datong University, Datong, China

**Keywords:** parental warmth, approaches to learning, self-efficacy, teacher-child closeness, moderated mediation analyses

## Abstract

Researchers have increasingly considered approaches to learning (ATL) a key indicator of school readiness. Our study purposed to examine the impacts of parental warmth on children’s approaches to learning, and the mediating role of self-efficacy, as well as the moderating role of teacher-child closeness in this relationship. Using a whole-group sampling method, 414 Chinese children aged 5–6 years participated this research together with their parents and teachers. Parents of those children were asked to fill out in person questionnaires on parental warmth, children’s approaches to learning, and self-efficacy. Children’s teachers completed the questionnaire regarding teacher-child closeness. Results indicated that children with high parental warmth were more likely to get high approaches to learning and their self-efficacy played a partial mediating role in this link. In addition, teacher-child closeness moderated the correlation between parental warmth and children’s self-efficacy. Specifically, the association between parental warmth and children’s self-efficacy was stronger for children with high teacher-child closeness than those with low teacher-child closeness. The results extend our understanding of how parental warmth affects children’s approaches to learning, revealing that strategies that could enhance self-efficacy would be effective in improving children’s approaches to learning.

## 1 Introduction

Researchers have increasingly considered approaches to learning (ATL) a key indicator of school readiness (Mcwayne et al., 2004) making ATL a research hotspot internationally (Repository and Vitiello, 2009; Blair and Razza, 2010; Bustamante et al., 2016). Children’s ATL are defined as learning-related dispositions, habits, attitudes, and styles that children demonstrate while engaging in the learning processes (Chen et al., 2011; Buek, 2019). These ATL include persistence and attentiveness, initiative, curiosity and interest, imagination and creation, as well as reflection and explanation (Yan, 2009). Early childhood is a critical period to form healthy habits and good behaviors (Yan, 2009), therefore, scholars from various contexts including the Republic of Korea (Lee and Lee, 2019) and China (Suo and Gao, 2020) have reached a consensus on the importance of ATL in relation to children. Morevoer, documents formulated by the governments worldwide have taken children’s ATL as one of developmental goals. For example, in the issued *The Goal 1 Technical Planning Subgroup Report on School Readiness* released by the American National Education Goals Panel (1991), children’s ATL were listed as one of the five foundational school readiness domains. Similarly, the *Statutory Framework for the Early Years Foundation*, enacted by the British Department for Education (2017), integrated children’s ATL into the development of seven major domains. Likewise, in the promulgated *Guidelines to the Learning and Development of Children 3 to 6* released by the Ministry of Education of the People’s Republic of China (2012), children’s ATL were emphasized as the essential quality for lifelong learning and development. Following the recognition from scholars and policy-makers, a growing body of literature has investigated the impact of children’s ATL on a wide range of their developmental results, including increasing cognitive ability, problem solving skills, resilience, and academic performance (Mcclelland et al., 2000; Mcwayne et al., 2004; Yen et al., 2004). Given the significant role of children’s ATL in their developmental results, further studies of factors that may contribute to children’s ATL are warranted. It may provide parents and teachers with insights into promoting strategies in a timely manner. It is also apparently that parenting style is imperative to various aspects of children’s development such as ATL (Jeynes, 2007; Ansari and Gershoff, 2016). While existing studies (Furrer and Skinner, 2003; Fantuzzo et al., 2007) have demonstrated the correlation between parenting style and children’s ATL, the ways in which parenting style affects children’s ATL in Chinese society and culture as well as the mechanisms driving this association are not fully understood.

The current paper attempts to add to the existing literature in two ways. First, there is a lack of empirical evidence regarding the possible relationships between parenting styles and children’s ATL in the Chinese context. Given the unique norms of the China’s preschool education system, the function of parenting styles in children’s developmental outcomes is influenced by obedience requirements and authoritarian management styles in kindergartens. Specifically, emotional and behavioral control at the early age is regarded as a prerequisite for effective learning (Ho, 2014). Accordingly, authoritarian teachers typically maintain classroom disciplines to achieve this (Cheng, 1996; Ng and Rao, 2008). An emphasis on disciplines is unfavorable for children’s problem-solving skills (Biggs, 1996; Ng and Rao, 2008), flexibility, independent learning, and even hampers their creativity (Murphy, 1987). Children from kindergartens spend a large amount of time engaging in interaction with teachers (Ferreira et al., 2016). Therefore, their habits and behaviors cultivated at home may be reshaped by preschool features. In addition, little is currently known about the potential role of mediators and moderators playing on the links between parenting style and children’s ATL. Particularly, there is a need to examine individual psychological traits (e.g., self-efficacy) as mediators (Lee and Lee, 2019) and the environmental factors (e.g., teacher-child relationship) as moderators (Sabol and Pianta, 2012) in these relationships. Given these gaps, the major aim of this study was to investigate the underlying mechanisms of the relationships between parenting style and ATL in children utilizing a sample of Chinese children from kindergartens.

## 2 Literature review

### 2.1 Parental warmth and children’s ATL

Differences in parenting styles yield different results in children’s development (Talib et al., 2011). Maccoby and Martin’s two-dimensional model states that the combination of the two main parenting dimensions (i.e., warmth and strictness) gives rise to four parenting styles: authoritative (warmth and strictness), neglectful (neither strictness nor warmth), indulgent (warmth but not strictness), and authoritarian (strictness but not warmth) (Maccoby and Martin, 1983). Parental warmth, a key dimension in these styles, is characterized by parents’ love, emotional support, positive affect, and acceptance to their children, as well as the involvement in schooling (Rohner and Britner, 2002; Rohner, 2004). The ecosystem theory proposes that family, as the micro system of children’s development, is an essential basis for their interaction with the outside world. Parenting styles have a key influence on children’s developmental outcomes, including habits, intelligence, ability, personality, interests, attitudes, and cognitive styles (Bronfenbrenner and Morris, 2006; Wang, 2017). Empirically, a meta-analysis involving 52 studies confirmed the impact of parental involvement on students’ academic attitudes and behaviors (Jeynes, 2007). Research conducted among American children (Ansari and Gershoff, 2016) and adolescents (Smalls, 2010) illustrates that parental warmth had a positive influence on children’s motivation, attention, persistence, interests, and active participation in class, with studies among Chinese children yielding similar results (Yang, 2019). Based on the above empirical evidence, the following hypothesis was proposed:

*Hypothesis 1*: parental warmth would be positively correlated with children’s ATL.

### 2.2 The mediating role of self-efficacy

Self-efficacy refers to the belief an individual has in their ability to accomplish a specific task under specific conditions (Bandura and Watts, 1996). In line with attachment theory, parenting styles affect various aspects of children’s development, including self-efficacy (Bretherton, 1985). Authoritative parenting-of which offering warmth is the core feature-prompts children to complete tasks and obtain high scores (Quach, 2008; Baker, 2017). This result is also linked to self-efficacy owing to the reciprocal effect of good academic performance and self-efficacy (Bandura and Watts, 1996). Empirical research conducted in American high school students (Choi et al., 2015) and children (Nokali et al., 2010; Ice and Hoover-Dempsey, 2011) found that parents’ support, acceptance, and participation positively influence their self-efficacy. Studies undertaken among Israeli college students (Alt, 2015) and Australian children (Reid et al., 2015) found that parental warmth develops creative self-efficacy through creating a good environment, with studies among Chinese primary school students yielding similar results (Zhang et al., 2018).

As per the self-efficacy theory (SET), self-efficacy determines whether coping behavior will be intuitively used, and how long individuals will persist facing up to obstacles (Bandura and Watts, 1996). It is involved in regulating children’s initiative and persistence that comprise ATL. Empirical study conducted among teachers in Turkey found that self-efficacy encourages individuals to use learning strategies creatively when carrying out a task and actively respond to setbacks (Erozkan, 2014). Research conducted in the Republic of Korea (Kim, 2011; Lee and Lee, 2019) and the USA (Yi and Hwang, 2003) also reports the positive predicting effect of self-efficacy on ATL, while study conducted among Chinese children produces a similar conclusion (Lin and Ye, 2020). Based on the above empirical studies, the following hypothesis was proposed:

*Hypothesis 2*: self-efficacy plays a mediating role between parental warmth and children’s ATL.

### 2.3 The moderating role of teacher-child closeness

Although parental warmth was linked with self-efficacy in several studies, not all children who experience low parental warmth demonstrate low general self-efficacy (Gong and Cao, 2018). A review of the existed study found that teacher-child relationship might explain this inconsistent finding.

Teacher-child relationship variables consist of closeness, dependency, and conflict (Vick, 2008). Teacher-child closeness is conceptualized by affection, warmth, and open communication between teacher and children (Pianta and Steinberg, 1992), directly promoting children’s social emotion and interaction behaviors. This may lead to children feeling safe in spontaneous exploratory behaviors and confidently taking the initiative to discuss their opinions with their teachers (Li, 2019), which may result in an enhancement of academic performance and an increase in self-efficacy (Suntheimer and Wolf, 2020). The social cognitive theory (SCT) posits that individuals’ self-efficacy is influenced by feedback from significant others and good interpersonal relationships (Bandura, 1977). As significant others, teachers positively impact children’s self-efficacy due to their role in guiding, supporting, and maintaining a close relationship with children (Yoon, 2002). Empirical studies from the USA illustrate that children who feel close to their teachers are more likely to attain better academic achievements, helping them gain high self-efficacy, than those who report adverse teacher-child relationships (Silver et al., 2005). Research conducted among American middle school students (Sakiz et al., 2012) and children (Vanoss, 2013) found a direct impact of close teacher-child relationships on the cultivation of children’s self-efficacy. Similar results have been obtained from research undertaken in China (Gu et al., 2017).

Moreover, teacher-child closeness moderates the relationship between parental warmth and self-efficacy. Protective-protective model states the predictive function of one protective factor (parental warmth) on outcome variable (self-efficacy) varies with the level of another protective factor (teacher-child closeness) (Brook et al., 1986). The multiple attachment hypothesis postulates that children’s multiple attachment relationships to parents and teachers have converging influences (Goossens and Ijzendoorn, 2010). Empirical studies among children (Sabol and Pianta, 2012) and adolescents (Rhodes and Resch, 2000) from the USA found that a close teacher-child relationship accelerates the advantageous influence of favorable family factors on children’s development, while research with Chinese children also indicates that close teacher-child relationships boost the influence of supportive parenting on children’s social skills (Wang et al., 2021). Based on the above empirical studies, the following hypothesis was proposed:

*Hypothesis 3*: teacher-child closeness moderates the beneficial effects of parental warmth on self-efficacy, and the association between parental warmth and self-efficacy enhances with the increase of teacher-child closeness.

**Figure 1:**
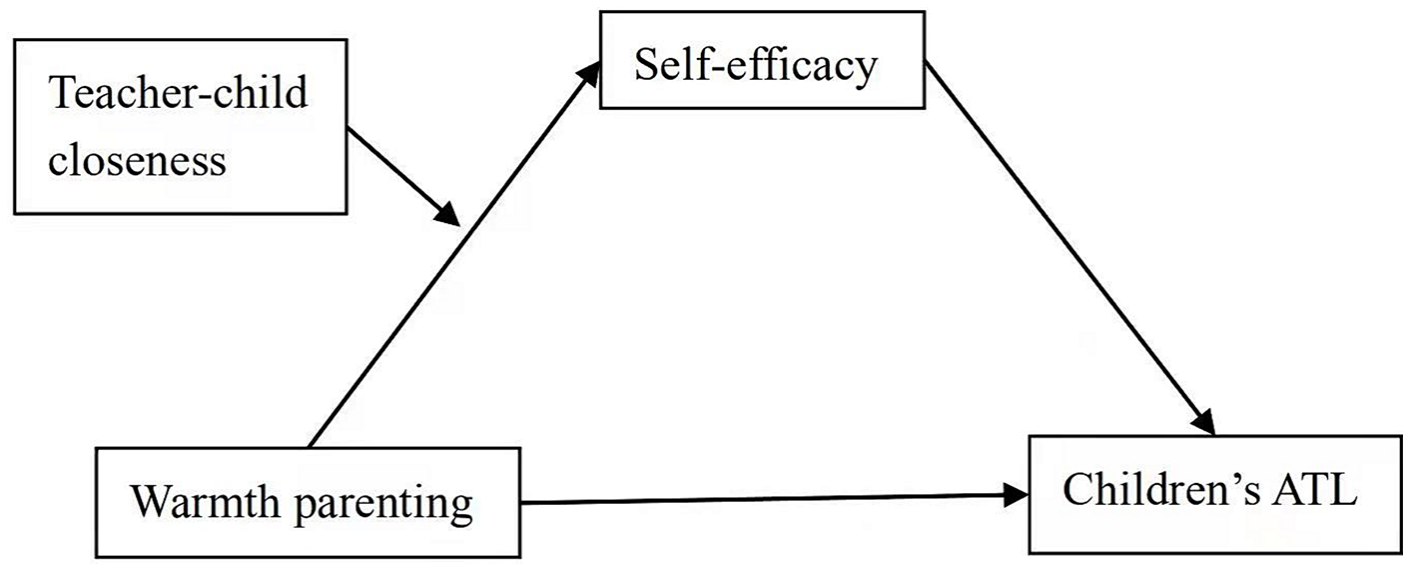
The proposed moderated mediation model.

Taken together, the conceptual model of this study is illustrated in Figure 1.

## 3 Methods

### 3.1 Participants

The data was collected from Datong, Shanxi province, China. This study adopted whole-group sampling method to target subjects. According to the China’s preschool school education system, children in K3 (i.e., the third-year preschoolers) are mostly aged 5–6 years old. Therefore, this study takes all children from intact K3 classes as investigated subjects. 14 intact K3 classes were selected from 6 kindergartens. The subjects consist of 198 boys (47.83%) and 216 girls (52.17%). Children’s homeroom teachers and one of their parents participated in the current study by filling a structured questionnaire. A sample of 414 questionnaires was returned. Parents’ educational background distribution was as below: high school (198, 47.8%), junior college and bachelor degree (108, 26.1%), lower than high school (72, 17.4%), and college degree or above (36, 8.7%). The mean per-capita income of family was as follows: 18 (4.3%) less than two thousand dollars (inclusive), 288 (69.6%) two-five (inclusive) thousand dollars, 90 (21.7%) five-ten (inclusive) thousand dollars, and 18 (4.3%) over ten thousand dollars.

### 3.2 Measures

#### 3.2.1 Parental warmth

Parents rated parental warmth with the Chinese version of the short-form Egna minnen av. Barndoms uppfostran (s-EMBU-C; Jiang et al., 2010). Indices of reliability for split-half reliability, Cronbach’s *α*, test–retest estimates, and the construct validity have been proved (Jiang et al., 2010). The s-EMBU-C contains two subscales, namely parental warmth and rejection. According to the actual need of this study, only warmth dimension was extracted from this scale. The warmth subscale is a 7-item (e.g., “I will praise my children”) instrument rated on a 4-point scale, which range from 1 (never) to 4 (always), to estimate a parent’s emotional warmth to his or her child. Average scores were calculated, with higher scores indicate a higher level of parental warmth. In this study, for the warmth dimension, McDonald’s omega was 0.94 and Cronbach’s *α* was 0.93.

#### 3.2.2 ATL

ATL were measured by the questionnaire of Children’s ATL (Cai, 2015). Consistent with its definition, Children’s ATL questionnaire contains five sub-dimensions, namely persistence and attentiveness (e.g., “Be able to persist in a good habit for a long time such as brushing teeth or reading every day”), initiative (e.g., “Proactively approach new things”), curiosity and interest (e.g., “Be interested in natural phenomena, such as thunder, rain, and plant growth”), imagination and creation (e.g., “Be able to combine materials in new ways when building blocks”), as well as reflection and explanation (e.g., “Be able to recognize his/her mistakes and explain the reasons”). The 41-item instrument has been proved to have high validity and credibility (Liu, 2022). All the questions are positive statements of children’s ATL, which are filled in by parents based on their children’s usual performance. Parents reported on a 4-point Likert scale the extent to which each item applied to their child (1 = never to 4 = always). Average scores were computed, with higher scores indicate a higher level of children’s ATL. In this study, McDonald’s omega was 0.91 and Cronbach’s *α* was 0.90.

#### 3.2.3 Self-efficacy

Parents rated their children’s self-efficacy with Questionnaire for Evaluating Self-efficacy in Young Children (Teacher/Parent Version) (Huang and Yang, 2015), which was revised based on Chinese version of General Self-Efficacy Scale (Zhang and Schwarzer, 1995). The revised questionnaire was filled in by teachers or parents. One of the examples of revising is changing “I am confident that I can effectively cope with any sudden situation” to “My children will encourage himself/herself by saying ‘I can do it’ or ‘no problem’ when facing new problems or unexpected situations.” The validity of this scale has been proved (Lin and Ye, 2020). This scale is a 6-item (e.g., “If get in trouble, my children will deal with it by themselves instead of immediately asking for help”) instrument rated on a 5-point scale, which range from 1 (definitely does not comply) to 5 (fully comply). Items were averaged, with higher scores indicate a higher level of self-efficacy. In this study, McDonald’s omega was 0.73 and Cronbach’s *α* was 0.69.

#### 3.2.4 Teacher-child closeness

Teachers assessed their relationships with the target children using the Student-Teacher Relationship Scale (STRS; Pianta and Steinberg, 1992). The validity of this scale has been proved (Hou et al., 2019). This STRS consists of three subscales, namely closeness, dependence, and conflict. According to the actual needs of this study, only closeness was adopted. Closeness subscale assessed teacher’s feelings of affection with child (e.g., “The relationship between me and this child is intimate”). This subscale is a 7-item instrument rated on a 5-point scale (1 definitely does not apply; 5 definitely apply). Item responses were averaged, with higher scores indicate a higher level of teacher-child closeness. In this study, McDonald’s omega was 0.84 and Cronbach’s *α* was 0.83.

### 3.3 Procedure

This study obtained approval from the Research Ethics Committee of the first researcher’s University. The questionnaires were distributed in October of 2019. The anonymity of this study was stressed before collecting data.

A meeting was conducted to guide the directors of target kindergartens regarding the research objective in order to get their permission in data collection. The administration of measurement was conducted by trained assistants majoring in preschool education. The assistants distributed questionnaires, combined with a consent form, to parents when they picked up their children after classes. Parents passed the completed questionnaires to homeroom teachers when sending their children to kindergarten the next day. Likewise, homeroom teachers signed informed consent and returned the questionnaire to the assistants after finishing all the questions. The questionnaire consists of four parts, among which, parental warmth questionnaire, children’s ATL questionnaire and children’s self-efficacy questionnaire were completed by parents, while teacher-child closeness questionnaire was completed by homeroom teachers.

### 3.4 Data analyses

All data were dealt in SPSS 20.0. Raw scores were transformed to z-scores before analyzing. First, descriptive statistics and Pearson correlations were obtained to explore the relationships among parental warmth, children’s ATL, children’s self-efficacy and teacher-child closeness. Second, Hayes’s (2013) PROCESS macro (i.e., a widely used tool for testing mediation and moderation) (Model 4) was applied to estimate whether self-efficacy mediated the link between parental warmth and children’s ATL. Third, PROCESS (Model 7) (Hayes, 2013) was applied to test whether teacher-child closeness moderated the link between parental warmth and self-efficacy. Fourth, procedural control and statistical testing were applied to decrease and test common method deviation problem. Procedural control included informing the anonymity to the respondents (Zhou and Long, 2004). Confirmatory factor analysis was used to examine the common method deviation (Liu et al., 2019). In addition, the bias-corrected nonparametric percentile bootstrap method was used to test mediation effect. 5,000 bootstrapped samples were generated to estimate the confidence interval (CI) on the base of the original sample (*n* = 414), and 95% CI without zero indicated significant mediation effect. What is more, gender and monthly family income were included in the analyses as control variables.

## 4 Results

### 4.1 Preliminary results

The results of confirmatory factor analysis showed the fit indexes was low (χ^2^/df = 34.23, RMSEA = 0.28, CFI = 0.66, TLI = 0.57, GFI = 0.67, AGFI = 0.48, NFI = 0.66). Therefore, there is no serious common method deviation. Table 1 showed the means, standard deviations, and the correlations among variables. All study variables were significantly correlated with each other.

**Table 1:** Means, standard deviations, and correlation coefficients of all variables.

	M	SD	1	2	3	4
1. Parental warmth	3.80	1.02	1			
2. ATL	3.54	0.63	0.67^***^	1		
3. Self-efficacy	3.71	0.56	0.61^***^	0.59^***^	1	
4. Teacher-child closeness	3.81	0.71	0.65^***^	0.70^***^	0.70^***^	1

^***^p < 0.001, ^**^p < 0.01, ^*^p < 0.05, the same below.

### 4.2 The mediating effect of self-efficacy

In Hypothesis 2, the current study expected that self-efficacy would mediate the association between parental warmth and children’s ATL.

As Table 2 illustrates, (1) parental warmth was positively associated with children’s ATL, *b* = 0.63, *p* < 0.001 (Model 1). Hence, hypothesis 1 was supported. (2) parental warmth was positively associated with self-efficacy, *b* = 0.59, *p* < 0.001 (Model 2). (3) self-efficacy was positively associated with children’s ATL, *b* = 0.30, *p* < 0.001 (Model 3), and the correlation between parental warmth and children’s ATL was still significant (*b* = 0.41, *p* < 0.001). Bootstrapping indicated the significant mediation effect of self-efficacy in explaining the correlation between parental warmth and children’s ATL (indirect effect = 0.18, 95% CI = [0.11, 0.25]). These findings indicated that self-efficacy played a partial mediated role between parental warmth and children’s ATL. Hence, hypothesis 2 was supported.

**Table 2:** The mediation role of parental warmth.

Predictors	Model 1 (ATL)		Model 2 (Self-efficacy)		Model 3 (ATL)	
	*b*	*t*	*b*	*t*	*b*	*t*
Gender	0.83	13.48^***^	−0.04	−0.47	0.84	14.80^***^
Monthly family income	0.26	5.06^***^	0.00	−0.07	0.26	5.51^***^
Parental warmth	0.63	18.44^***^	0.59	19.24^***^	0.41	11.41^***^
Self-efficacy					0.30	8.49^***^
*R^2^*	0.63		0.38		0.68	
*F*	229.57^***^		82.07^***^		220.08^***^	

^***^p < 0.001, ^**^p < 0.01, ^*^p < 0.05.

### 4.3 Testing for moderated mediation

In Hypothesis 3, the present study presumed that the correlation between parental warmth and self-efficacy was moderated by teacher-child closeness.

As Table 3 illustrates, the correlation between parental warmth and self-efficacy was significant (*b* = 0.49, *p* < 0.001). More importantly, the interaction coefficient between parental warmth and teacher-child closeness was significant, *b* = 0.27, *p <* 0.001, indicating that the link between parental warmth and self-efficacy (the former half path of the mediation process) was moderated by teacher-child closeness. Hence, hypothesis 3 was supported.

**Table 3:** The moderated mediation effect of parental warmth on ATL.

Predictors	Model 1 (Self-efficacy)		Model 2 (ATL)	
	*b*	*t*	*b*	*t*
Gender	−0.32	−4.71^***^	0.84	14.80^***^
Monthly family income	−0.07	−1.33	0.26	5.51^***^
Parental warmth	0.49	9.75^***^	0.41	11.41^***^
Self-efficacy			0.30	8.49^***^
Teacher-child closeness	0.48	11.15^***^		
Parental warmth x Teacher-child closeness	0.27	7.62^***^		
*R^2^*	0.60		0.68	
*F*	121.87^***^		220.09^***^	

^***^p < 0.001, ^**^p < 0.01, ^*^p < 0.05.

**Figure 2:**
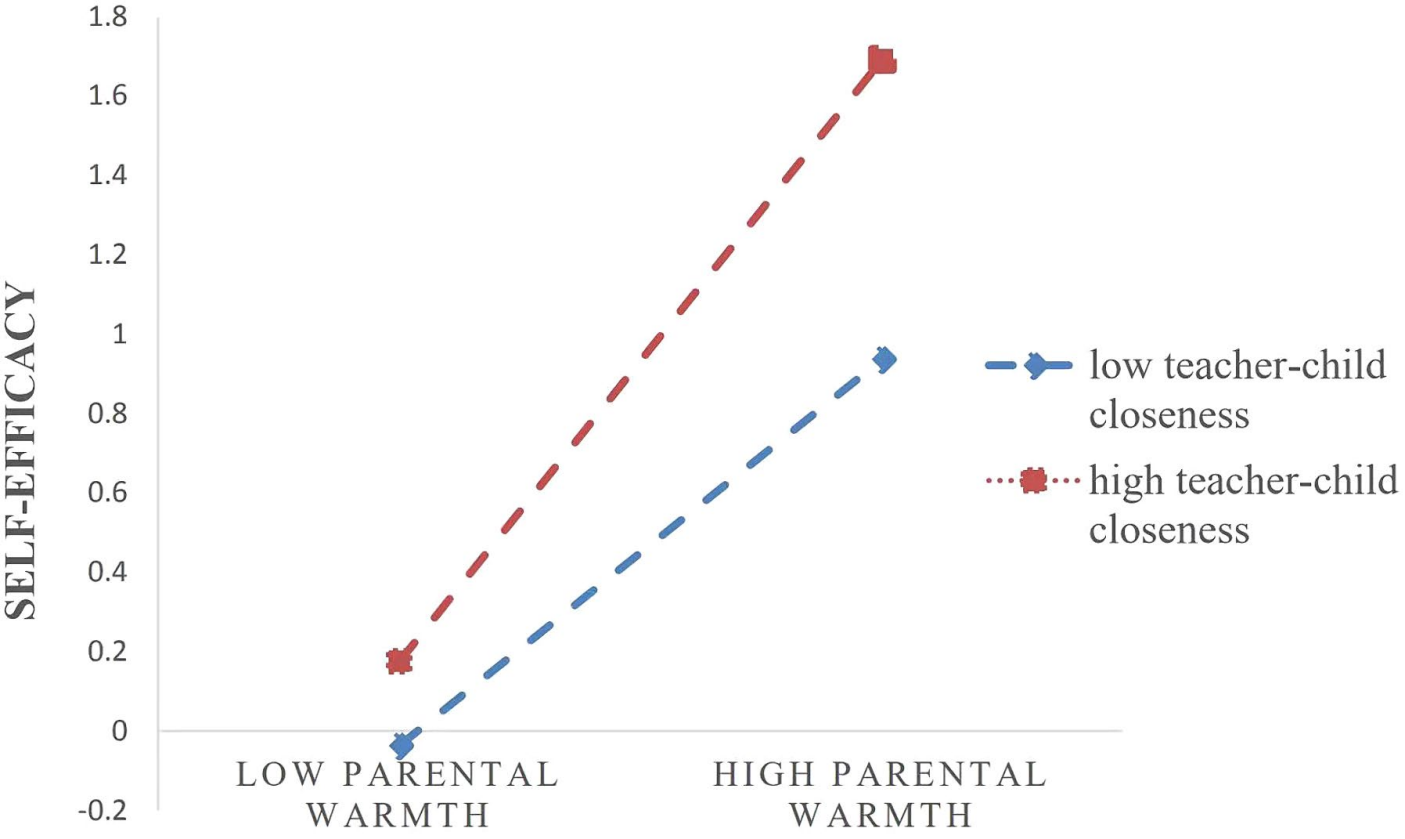
Teacher-child closeness as a moderator of the link between parental warmth and self-efficacy.

To test the interpretation of the interaction effect, this study plotted regression lines separately for high and low teacher-child closeness (1 SD above and 1 SD below the mean, respectively) (Figure 2). Simple slope tests showed that for children with high teacher-child closeness, higher levels of parental warmth were associated with higher levels of self-efficacy (*b*_simple_ = 0.76, *t* = 10.00, *p* < 0.001). However, for children with low teacher-child closeness, the link between parental warmth and self-efficacy was still significant but much weaker (*b*_simple_ = 0.21, *t* = 5.10, *p* < 0. 001).

## 5 Discussion

The current study empirically utilized a sample of children from China and tested three research hypotheses. This section outlines the major findings discussed alongside related literature.

### 5.1 The impact of parental warmth on children’s ATL

The current study indicated that parental warmth was positively associated with children’s ATL, thus supporting hypothesis 1. This finding is consistent with the existing finding in the USA (Furrer and Skinner, 2003), in which parental warmth was positive related with children’s participation in class. The constructivist theory of learning indicates that parenting styles that taking children as the center of knowledge construction would promote the learning and development of children (Lin and Ye, 2020). That is to say, parental support and encouragement affect children’s behavior and attitude in learning processes (He, 1997).

### 5.2 The mediating role of self-efficacy

Meditational analyses revealed that self-efficacy was an important mediation variable in the correlation between parental warmth and children’s approach to learning, thus supporting hypothesis 2. Similarly, empirical research conducted among Chinese adolescents indicates that math self-efficacy arise from the increase of paternal warmth leads to a higher level of math engagement (Sun et al., 2020). According to the self-determination theory (SDT), intrinsic learning motivation originates in the satisfaction of psychological needs (e.g., sense of belonging, sense of autonomy, and sense of ability), the fulfillment of which will motivate individuals to learn initiatively, be curious about learning, and persist in learning (Deci and Ryan, 2000; Ryan and Deci, 2000). Specifically, parental warmth meets children’s psychological needs through providing love and support, encouraging independence of thoughts, as well as giving opportunities for children to do things independently (e.g., “If my children are faced with a difficult task, they can feel my support and love for them” “Even if my children’s views are different from mine, I still encourage them to express themselves freely and respect their decision to do things,” and “I encourage my children to do things alone”). The satisfaction of psychological needs empowers children to learn with initiative, curiosity, and persistence.

What is more, it is worth explaining each of the separate associations in the mediation model. For the first stage of the mediating process, this finding echoes the previous study from Thailand (Aumeboonsuke, 2017), Turkey (Bingöl, 2018), and the USA (Oliver and Paull, 1995) in which parents’ emotional warmth helps children develop a high level of self-efficacy. According to the self-efficacy theory, family and various social factors are one of four sources of self-efficacy. Parents’ support, acceptance, and encouragement in various forms help children build confidence in their abilities (Bandura and Watts, 1996). For the second stage of the mediation process, this finding is aligned to previous research from Singapore (Liem et al., 2008) and the USA (Zimmerman et al., 2016), in which self-efficacy influences learners in initiative, endeavor, persistence to study, and reflections for learning results, all of which are considered central constituents of ATL.

### 5.3 The moderating role of teacher-child closeness

The current study revealed that the protected effect of parental warmth on children’s self-efficacy would be further mitigated by teacher-child closeness, which verifies hypothesis 3. Similarly, empirical studies from Belgium (Buyse et al., 2011) and the USA (Meehan et al., 2003) have shown that teacher-child relationship is an essential moderation mechanism in the association between parent–child relationship and children’s problem behavior. This finding could be elucidated by the overlapping spheres of influence theory, which indicates that school, family, and community affect children’s development independently or jointly (Yang, 2006; Epstein, 2010; Tang, 2019). The participation of teachers makes the link among the three spheres closer (Pianta and Walsh, 1996). Teacher-child closeness regulates the effect of parental warmth on children’s positive outcomes by providing a cognitive representation of parent–child relationship (O’Connor and McCartney, 2007; Mortensen and Barnett, 2015; Tang, 2019). Notably, the support and help from teachers is conducive for children to trust and accept the love, support, and encouragement from their parents (Ryan et al., 1994; Epstein, 1998), which may shape their representations of self. This may also lead children to perceive themselves as worthy, and thus obtain affirmation of their own abilities (Weyns et al., 2019). In other words, a close teacher-child relationship propels children who experience parental warmth to exhibit higher self-efficacy.

### 5.4 Implications

First, the findings of this study would be helpful for researchers to integrate related theories as a framework in predicting children’s ATL. Theoretically, the current study constructed a moderated mediation model to clarify the influencing factors and underling mechanism of children’s ATL, relying on ecosystem theory, attachment theory, self-efficacy theory (SET), and social cognitive theory (SCT). However, the theoretical framework for these quantitative summaries and applied theories are scattered and vague. Proposing a conceptual framework would assist practitioners, academics, and students to find the associations between children’s ATL and their antecedents. Otherwise, they need to synthesize the contents of a body of empirical and conceptual papers.

Second, the present study provides practical inspiration for the practitioners with crucial insight on children’s care. First, parents training can help parents change the way of interacting with their children, and turn parenting control into parental warmth. However, current education programs put less emphasis on parents education in China (Zhang et al., 2008). Besides, parents should pay special attention to the cultivation of children’s self-efficacy. When judging the success or failure of education, they need to take the improvement of children’s self-efficacy as an important basis rather than just focusing on the acquisition of knowledge, skills or achievement. For example, they can offer specific, frequent, and immediate feedback to children regarding their daily performance (Weiser and Riggio, 2010). Apart from that, educators can also develop children’s self-efficacy during play (Kermani and Younesi, 2014). Strategies include encouraging them to set the rules of group games on their own and teaching them peer communication skills to strengthen their cooperation with the group.

Furthermore, the findings of this study would be helpful for the policy-makers to formulate related projects. Projects that combine parents’ education with teachers’ education would be more efficient than those only focusing on one group as the current study shows teacher-child closeness regulates the effect of parental warmth on children’s developmental outcomes. Since China has a long history of discrete and separate working relationships between teachers and parents (Guo and Kilderry, 2018), a mix teacher-parent partnership needs to be special emphasized in early childhood policies.

### 5.5 Limitations and future directions

These findings suggest five directions for future research. First, although the mediation analyses showed that children’s self-efficacy played a mediation role, self-efficacy may serve as a “common outcome” of parental warmth and teacher-child closeness. Theoretically, it is rational because positive relationships with teachers (Ye, 2007) and parents (Venden, 2004; Coleman, 2010; Yuan et al., 2016) all foster children’s self-efficacy. Therefore, more refined longitudinal researches were useful to examine which process better accounts for the association. Second, a teacher rated his/her relationships with multiple children in their classes through self-report instrument, which may lead to high intra-class correlations and social desirability bias in the data. Alternative methods such as longitudinal studies and experimental manipulation should be carried out in the future to help us make strong assertions about causality between variables. Third, the samples could not stand for the diversity of Chinese society as they were drawn from a middle city in China. Therefore, more diverse samples need to be drawn to clarify whether the pattern is unique to Chinese children or universal to all children from different backgrounds. Forth, this study only investigated parents neglecting other main caregivers (e.g., grandparents). As more and more children are adopted by their grandparents especially in big cities and the parenting styles between parents and grandparents differ greatly (Birmingham et al., 2021), analyzing intergenerational difference is of great practical significance. Finally, preschool children’s ages are theoretically different but only one age group was measured in the present paper, it will be meaningful to target children of different ages.

### 5.6 Conclusion

The current study aimed to offer insights into how (self-efficacy as a mediator) and under what conditions (teacher-child closeness as a moderator) parental warmth could promote children’s ATL. Research found that parental warmth could influence children’s ATL through the mediating role of self-efficacy, which was moderated by teacher-child closeness. The association was stronger for children with high teacher-child closeness than for their low teacher-child closeness counterparts. Based on these findings, strategies that could enhance self-efficacy would be effective in improving children’s ATL. Results also demonstrated that school teachers play a significant role in working with families to accelerate children’s self-efficacy at an early age.

## Data availability statement

The raw data supporting the conclusions of this article will be made available by the authors, without undue reservation.

## Ethics statement

The studies involving humans were approved by this study was conducted in line with the Declaration of Helsinki. The study got the approval of the Research Ethics Committee of Shanxi Datong University. The studies were conducted in accordance with the local legislation and institutional requirements. Written informed consent for participation in this study was provided by the participants’ legal guardians/next of kin.

## Author contributions

YL: Writing – original draft. WW: Writing – review & editing. SW: Data curation, Writing – review & editing. PW: Conceptualization, Writing – review & editing. KC: Methodology, Writing – review & editing. JL: Formal analysis, Writing – review & editing. JC: Writing – review & editing.
